# A novel predictive factor for the onset time of docetaxel-induced onychopathy: a multicenter retrospective study

**DOI:** 10.1186/s40780-016-0057-4

**Published:** 2016-09-29

**Authors:** Hidenobu Takahata, Kouichi Tanabe, Akiyoshi Takaki, Tsuneaki Yamanouchi, Yasuhiko Mimura, Atsumi Nitta, Hatsuna Yasuda, Tatsuhiko Kashii, Isao Adachi

**Affiliations:** 1 Department of Pharmacy, Tonami General Hospital, Tonami, Japan; 2 Department of Pharmacy, Toyama University Hospital, Toyama, Japan; 3 Department of Pharmaceutical Therapy and Neuropharmacology, Faculty of Pharmaceutical Sciences (Pharmacology), Graduate School of Medicine and Pharmaceutical Sciences, University of Toyama, Toyama, Japan; 4 Department of Medical Oncology, Toyama University Hospital, Toyama, Japan; 5 Drug Informatics, Faculty of Pharmacy, Meijo University, Nagoya, Japan

**Keywords:** Docetaxel, Taxane, Chemotherapy, Adverse event, Prediction

## Abstract

**Background:**

Taxanes are known to cause onychopathy. Previous studies have reported the relationship between onychopathy and paclitaxel dosing intervals and cumulative doses. However, there are no studies of the predictive factors for docetaxel-induced nail changes. The present study used the drug accumulation rate (mg/m^2^/day) as a novel indicator and evaluated its usefulness for the prediction of onychopathy.

**Methods:**

From January 2008 to December 2009, we examined patients who received docetaxel at the Toyama University Hospital and Tonami General Hospital to determine the time to onset of onychopathy, the accumulation rate, and the cumulative dose. We then divided the study subjects into two groups, and used Receiver Operating Characteristic (ROC) analysis to calculate a cut-off value. We evaluated both indicators as predictive factors for onychopathy using the log-rank test and Cox proportional hazards model.

**Results:**

Ninety-five patients were included in the present study. The results of the log-rank test sub-analysis revealed that the median number of days until onychopathy onset was significantly shorter in patients with an accumulation rate greater than the cut-off (*P* = 0.009), and in those with a cumulative dose below the cut-off (*P* < 0.001). The hazard ratios for the accumulation rate and cumulative dose, evaluated using Cox proportional hazards regression analysis, were 1.44 (*P* = 0.036) and 0.99 (*P* < 0.001), respectively.

**Conclusions:**

The results of the present study indicated that the drug accumulation rate influenced the time to onset of docetaxel-induced onychopathy.

**Trial registration:**

This study is not applicable for trial registration due to retrospective chart review without intervention.

## Background

Skin disorders caused by anticancer drugs are known to significantly affect quality of life when they become serious [1]. However, there may be differences in the evaluation of the severity of these symptoms by patients and medical practitioners. In addition, the concordance rate between these two types of evaluation also changes, depending on when they are performed. In particular, under-reporting of onychopathy by medical practitioners during clinical trials of anticancer drugs has been identified as a problem [2]. Therefore, it is important to determine the time of onychopathy onset, in order to perform accurate evaluations in a timely manner [3].

Onychopathy is a skin disorder that is known to impact on patient quality of life. The results of a clinical trial comparing weekly and tri-weekly administration of paclitaxel (PTX) indicated that onychopathy may arise due to the administration interval and the cumulative dosage, rather than occurring in response to a single dose [4]. Therefore, the exposure frequency and duration of use were thought to determine the risk for onset of onychopathy in patients receiving docetaxel (DTX), which is another taxane anticancer drug [5].

On the other hand, doxorubicin (like DTX) is a concentration-dependent drug and its accumulation rate has been identified as a new predictive indicator for the time of cardiotoxicity onset [6]. The accumulation rate may therefore provide a predictive factor for the onset of onychopathy in patients receiving DTX. If this point can be clarified, it could improve the accuracy of predictions of the time of onychopathy onset, thus facilitating the establishment of more suitable intervention plans.

The present study focused on cumulative DTX dosage and accumulation rate in order to evaluate their utility as predictive factors for the time of onychopathy onset.

## Methods

### Study design

This multicenter retrospective cohort study was performed after having obtained the approval of the Toyama University Hospital Institutional Review Board (approval no. 126) and the Tonami General Hospital Institutional Review Board (approval no. 26003).

### Subjects

The subjects were patients administered DTX in the 2 years from January 2008 to December 2009 at the outpatient chemotherapy center of Toyama University Hospital and at the outpatient chemotherapy department of Tonami General Hospital. The observation period was from the date of DTX administration until the date that DTX treatment was discontinued. The performance status of the study subjects was 0 or 1. Furthermore, patients with onychopathy of Grade 1 or higher at the start of DTX administration were excluded from the study, as were patients with a history of DTX administration within the past 3 months.

### Investigation methods

Outcome: The primary outcome was the onset of ≥ Grade 1 onychopathy, diagnosed in accordance with the Common Terminology Criteria for Adverse Events (CTCAE) version 3.0.

Determination of onychopathy: The nails of both the hands were observed by the pharmacist in charge or by the nurse at the patients’ outpatient visits. The grade of onychopathy was determined using the CTCAE and entered into each patient’s electronic chart.

Investigation items: For each subject, we recorded the current regimen name, sex, age, performance status, body surface area, underlying diseases, start-date for DTX administration, end-date of DTX administration, date of onychopathy onset, grade, name of previous regimen, interval between previous and current regimens, number of days until onset of onychopathy, treatment period with DTX (days), mean administration interval, full cumulative dose of DTX (mg), use of several 5-FU derivatives (5-fluorouracil, capecitabine, tegafur/gimeracil/oteracil), and blood hemoglobin level (g/dL).

The DTX treatment period was defined as the time from DTX administration initiation to the first observation of onychopathy (for those who developed onychopathy) or to the pre-defined end-date of DTX treatment.

The mean administration interval was derived by dividing the number of DTX treatment days by the number of DTX administrations. The cumulative dose (mg/m^2^) was derived by dividing the sum of the DTX doses administered during the treatment period by the patient’s body surface area. The accumulation rate (mg/m^2^/day) was derived by dividing the cumulative dosage of DTX by the number of days of treatment with DTX.

### Statistical analysis

For the univariate analysis, cut-off values were calculated using the mean of each of the candidate predictive factors (cumulative dosage or accumulation rate), and these were used to divide the subjects into two groups: a High Group (Group H), at or above the cut-off value, and a Low Group (Group L), below the cut-off. Kaplan-Meier curves were plotted and Log-rank testing was performed in order to study the correlation between the cumulative dose of DTX and the accumulation rate with the number of days until the onset of onychopathy.

In addition, a multivariate analysis was conducted. The number of days until the onset of onychopathy was treated as the response variable, both the cumulative dose (mg/m^2^) and the accumulation rate (mg/m^2^/day) were treated as predictive factors, and Cox’s proportional hazard analysis was performed. Other variables that affect the onset of onychopathy, as well as nail growth (age, body surface area, presence of several 5-FU derivatives administration, and hemoglobin level) were selected as covariates and analyzed as descriptive variables, in tandem with the predictive factors.

## Results

### Subject characteristics

**Fig. 1:**
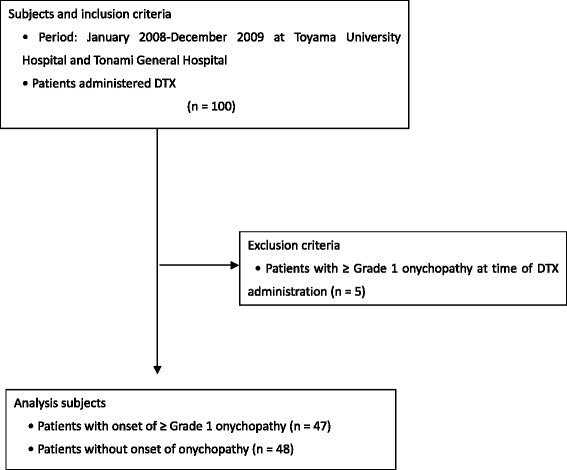
Flowchart of the participating patients. DTX, docetaxel

Ninety-five patients were administered DTX and also satisfied the inclusion criteria at the start of DTX administration; 47 of these exhibited onychopathy during the observation period (Fig. 1, Table 1). The mean cumulative dose and accumulation rate values calculated by ROC analysis were 112 mg/m^2^ and 2.1 mg/m^2^/day, respectively. The patients were divided into Group H and Group L on the basis of these cut-off values.

**Table 1:** Patient characteristics

Characteristics	Value (*n* = 95)	
Age (mean years ± S.D.)	62.1 ± 12.0	
Gender (*n*, male/female)	54/41	
Body surface area (m^2^, mean ± S.D.)	1.57 ± 0.17	
Concomitant use of 5-FU drugs^a)^ (*n*, Y/N)	33/62	
Hemoglobin at the start of administration (mean g/dL ± S.D.)	11.2 ± 1.8	
Primary site		
Stomach, *n* (%)	32	(33.7)
Breast, *n* (%)	23	(24.2)
Lung, *n* (%)	19	(20.0)
Prostatic, *n* (%)	9	(9.5)
Others, *n* (%)	12	(12.6)

^a)^5-FU drugs: 5-fluorouracil, capecitabine, tegafur/gimeracil/oteracil

In addition, 18 regimens had been used as previous treatments and 33 patients had used concomitant several 5-FU derivatives. The mean time from the previous treatment until the start of DTX administration was 112.5 ± 156.3 days (Table 2).

**Table 2:** Medication regimens of 47 patients who developed onychopathy

Regimen	Number	Previous regimens
DTX (60-80)/q3w	20	Carboplatin + PTX (3)
		Gefitinib (1)
		Carboplatin + gemcitabine (1)
		DTX (1)
		FEC (10)
		FP (1)
		Vinorelbine (1)
		Trastuzumab (1)
		XP (1)
DTX (70)/q3w/prednisolone	4	Goserelin (2)
		Leuprorelin (1)
		Estramustine (1)
S-1 (80)/cisplatin (40)/DTX (40)/q6w	10	None (10)
S-1 (80)/DTX (40)/q3w	3	Cisplatin + irinotecan (1)
		S-1 + cisplatin + DTX (1)
		None (1)
DTX (30)/qw	1	None (1)
DTX (20-40)/q2w	3	Tamoxifen + goserelin (1)
		PTX (1)
		None (1)
DTX (35)/qw/estramustine (560)	1	PTX + estramustine (1)
DTX (40)/q3w/carboplatin	4	Carboplatin + PTX (1)
		None (3)
Irinotecan (60)/DTX (35)/q2w	1	AP (1)

DTX, docetaxel; S-1, tegafur ∙ gimeracil ∙ oteracil; PTX, paclitaxel; FEC, epirubicin + cyclophosphamide + fluorouracil; FP, fluorouracil + cisplatin; XP, capecitabine + paclitaxel; AP, doxorubicin + paclitaxel; q3w, every 3 weeks; q6w, every 6 weeks; qw, every week; q2w, every 2 weeks.

### Correlation between the onychopathy onset time and each of the predictive factors (univariate analysis)

**Fig. 2:**
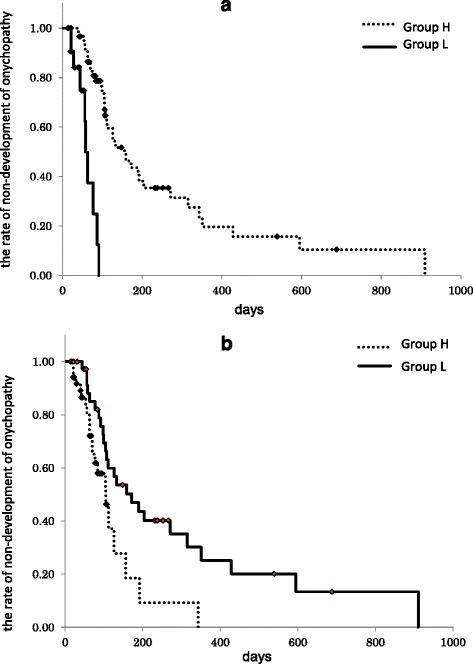
Curves showing the rate of non-development of onychopathy by cumulative dose (**a**) and accumulation rate (**b**). When the cut-off value for the accumulated dose was set at 112 mg/m^2^, the median value (time) before the onset of onychopathy was 156 days for Group H and 58 days for Group L (*p* < 0.001). When the cut-off value for the accumulation rate was set at 2.1 mg/m^2^/day, the median value (time) was 105 days for Group H and 172 days for Group L (*p* = 0.009)

Figure 2 (a) and (b) show the Kaplan-Meier curves for cumulative dose and accumulation rate respectively. Log-rank testing found that the median number of days until onychopathy onset was significantly shorter in patients classified as Group H by their accumulation rate (*P* = 0.009), and in those classified as Group L by their cumulative dose (*P* < 0.001) (Table 3).

**Table 3:** Correlation of onychopathy onset time with various predictive factors

Factor/Model	Univariate analysis^a)^			Multivariate analysis^b)^	
	Group H ^c)^	Group L ^c)^	*P* value	Hazard ratio (95 % CI)	*P* value
Accumulation rate (mg/m^2^/day)	104 (14-343)	159 (15-910)	0.024	1.4429 (1.0238, 2.0336)	0.0363
Cumulative dose (mg/m^2^)	204 (42-910)	77 (14-315)	<0.001	0.9905 (0.9862, 0.9948)	<0.001

### Cox’s proportional hazard analysis (multivariate analysis)

This analysis identified the hazard ratio (HR) for cumulative dose as a predictive factor, was 0.99 (*p* < 0.001), and the only significant factor among the other covariates was 5-FU-derivative administration (HR = 0.23, *p* < 0.001). The HR for accumulation rate as a predictive factor was 1.44 (*p* = 0.036), and the only significant factor among the other covariates was age (HR = 0.97, *p* = 0.03) (Table 3).

CI, confidence interval; ^a)^Log-rank test of the number of days until onychopathy onset, expressed as the median (range); ^b)^Cox’s proportional hazard analysis with age, body surface area, presence of several 5-FU derivatives administration, and hemoglobin (g/dL) as covariates; ^c)^Group H had accumulation rates of ≥ 2.5 mg/m^2^/day or cumulative dosages of ≥ 200 mg/m^2^. Group L had levels below these cut-off values.

## Discussion

This study implied that the DTX accumulation rate is an important determinant of the onset of DTX treatment-associated onychopathy. The onset of onychopathy induced by DTX reflects dose-dependent cumulative toxicity [7] and the accumulation rate also represents an important factor predicting the severity of this adverse event. Previous studies have shown wide variation in the time of onset of onychopathy; this has therefore been difficult to predict and tended to be overlooked, resulting in late diagnosis at a severe stage, and treatment by dosage reduction or discontinuation [8]. Minisini [4] and Takeshita et al. [9] reported that DTX-associated onychopathy was present at earlier time-points than those reported by clinical trials and emphasized that early treatment was important. It is anticipated that the effectiveness of in-hospital risk management plans will be improved by the use of more reliable predictive factors for the time of onychopathy onset. This onset time information will enable more attention to be directed to the nails in the period during the appropriate time-period, reducing the prevalence of treatment delays and of untreated onychopathy. In addition, this information will improve understanding of the cause/effect relationship between the causal drug and onychopathy, facilitating rapid action.

In the present univariate analysis of the cumulative dose, the number of days until onset of onychopathy was significantly lower in Group L. This may have been because of rather than the size of the cumulative dose affecting the time to onset of onychopathy, the fact that administration was discontinued after the appearance of onychopathy, resulting in a lower cumulative dose. On the other hand, univariate analysis of the accumulation rate identified significantly fewer days until the onset of onychopathy in Group H and multivariate analysis produced a hazard ratio of 1.44. The most considerable reason for that could be the reversibility of side effects. The longer drug holiday periods will improve recovery of the damage that cells received when the side effect is reversible.

Therefore it is necessary to consider ‘time’ as a factor in order to predict the onset of side effects in this case. On the other hand, the cumulative dose could directly effect on cumulative damage in the case of doxorubicin-induced cardiotoxicity and other side effects that are irreversible, which predictive index is lifetime cumulative dose no matter how long drug holiday periods are. The accumulation rate (mg/m^2^/day) used in the present study was very similar to the concept of dose intensity (mg/m^2^/week). However, narrowly-defined, dose intensity is often used as a theoretical measure of the intensity of a regimen, whereas the accumulation rate (mg/m^2^/day) allows for the inclusion of patient-specific dosage reductions and drug holiday periods. Therefore, the accumulation rate can provide a more useful indicator than dose intensity for drugs like DTX, which show a long intracellular retention time and high concentration-dependency [10].

The time to onset of onychopathy cannot be predicted by the accumulated dose alone. We believe that the accumulation rate is appropriate for predicting when onychopathy will appear. This is because the cut-off values calculated from ROC analysis for the conventional accumulated dose produced the paradoxical result that the median value (time) to the onset of onychopathy was longer in Group H, as shown in Fig. 2a.

This study had some noteworthy limitations. First, this was a retrospective investigation and a prospective cohort study will be necessary in order to verify the present results, including the causal relationship between the accumulation rate and onychopathy. Second, because the onset of onychopathy was determined on the date of the patient’s outpatient visit, there is a possibility that the actual onset occurred earlier and this was not necessarily accurate. And that is, the possibility that the appearance of onychopathy after the discontinuation of DTX administration was overlooked cannot thus be ruled out. Third, because the number of cases was limited and the effects of previous treatments were not taken into account in this study, verification will be necessary by studies that incorporate measures to minimize the effects of previous treatments; this could include studies of administration-naïve patients only, in order to obtain more accurate results. However, despite the above limitations, the implication that the accumulation rate could provide a novel predictive factor for the onset of DTX-associated onychopathy has important clinical ramifications. As described above, this approach could improve understanding of the cause and effect relationship between drug treatments and the onset of this condition, helping to reduce the level of undiagnosed onychopathy and minimize treatment delays.

Cancer chemotherapy is transitioning from an inpatient procedure to an outpatient treatment. Depending on the individual, there are cases where a patient does not recognize that a skin disorder has occurred as an adverse reaction to the anticancer drug. Even when the patient is aware of this, they often simply ignore it because they consider it to present a minor inconvenience. This study may also lead to further investigations of whether the accumulation rate is also a better predictor of other adverse reactions treated as cumulative toxicities, including cisplatin-induced ototoxicity and oxaliplatin-induced peripheral neuropathy [11].

## Conclusion

The present study identified the DTX accumulation rate as a possible new predictive factor for the onset of onychopathy. In the future, it will be necessary to verify the current results by conducting prospective observational trials, and to explore whether the predictive accuracy can be increased.

## Abbreviations

CTCAE: Common Terminology Criteria for Adverse Events
DTX: Docetaxel
5-FU: 5-fluorouracil
PTX: Paclitaxel
